# Predicting the Disease Risk of Protein Mutation Sequences With Pre-training Model

**DOI:** 10.3389/fgene.2020.605620

**Published:** 2020-12-21

**Authors:** Kuan Li, Yue Zhong, Xuan Lin, Zhe Quan

**Affiliations:** ^1^School of Cyberspace Security, Dongguan University of Technology, Guangdong, China; ^2^Guangdong Key Laboratory of Intelligent Information Processing, Shenzhen, China; ^3^Department of Computer Science, Xiamen University, Xiamen, China; ^4^College of Information Science and Engineering, Hunan University, Changsha, China

**Keywords:** BERT pre-training model, protein sequence, hydrophilicity, protein mutation, BRCA1 gene

## Abstract

Accurately identifying the missense mutations is of great help to alleviate the loss of protein function and structural changes, which might greatly reduce the risk of disease for tumor suppressor genes (e.g., BRCA1 and PTEN). In this paper, we propose a hybrid framework, called BertVS, that predicts the disease risk for the missense mutation of proteins. Our framework is able to learn sequence representations from the protein domain through pre-training BERT models, and also integrates with the hydrophilic properties of amino acids to obtain the sequence representations of biochemical characteristics. The concatenation of two learned representations are then sent to the classifier to predict the missense mutations of protein sequences. Specifically, we use the protein family database (Pfam) as a corpus to train the BERT model to learn the contextual information of protein sequences, and our pre-training BERT model achieves a value of 0.984 on accuracy in the masked language model prediction task. We conduct extensive experiments on BRCA1 and PTEN datasets. With comparison to the baselines, results show that BertVS achieves higher performance of 0.920 on AUROC and 0.915 on AUPR in the functionally critical domain of the BRCA1 gene. Additionally, the extended experiment on the ClinVar dataset can illustrate that gene variants with known clinical significance can also be efficiently classified by our method. Therefore, BertVS can learn the functional information of the protein sequences and effectively predict the disease risk of variants with an uncertain clinical significance.

## 1. Introduction

Function loss of the tumor suppressor gene BRCA1 (Chenevixtrench et al., 2006) results in the risk of breast and ovarian cancer in women (Hall et al., 1990). The most common variants of uncertain significance (VUSs) (Landrum et al., 2016) in the BRCA1 gene are single nucleotide variations (SNVs), which may lead to missense substitutions of amino acid. Among 1,863 amino acids of the BRCA1 gene, it is reported that 12,458 SNVs in these amino acids may potentially cause missense substitutions, so that it will further affect protein function (Starita et al., 2018). Once the protein function is affected by missense mutations, loss of BRCA1 activity results in the fact that cells fail to repair the broken DNA. Thus, being able to predict the missense mutation in proteins is of major significance to better understand the function of molecules and cells, and to reduce the risk of disease.

Traditionally, commonly used experimental methods, including the multiplex HDR (homology-directed DNA repair) reporter assay (Starita et al., 2018) and saturated gene editing (Findlay et al., 2018), prefer to classify the BRCA1 gene variants by measuring the function of HDR (Pierce et al., 1999). These methods are limited to specific biological functions of the corresponding genes. On the other hand, *in silico* methods for variant classification require prior knowledge of genetic variants such as refGene annotations (Pruitt et al., 2014), gnomAD (Lek et al., 2016), while many variants (i.e., VUSs) cannot be classified owing to the lack of prior knowledge. These experimental methods are both expensive and time-consuming.

Data-driven machine learning approaches can complement experimental methods and permit large-scale investigations (Jin et al., 2019; Su et al., 2019a,b). *Sequence-based* and *structure-based* methods are widely designed to learn the protein function and to solve problems in related tasks (Wei et al., 2018, 2019; Lin et al., 2019, 2020a). While structure-based methods are limited due to the unavailable 3D structures of most known proteins. Thus, protein engineering informatics provide better solutions for learning protein sequences and model the relationship between sequence and function (Romero and Arnold, 2009; Packer and Liu, 2015). Meanwhile, with the available datasets of protein sequences increasing exponentially (Alley et al., 2019), much machine learning methods have been devoted to learning from protein sequence (Zou et al., 2019). For example, ProtVec (Asgari and Mofrad, 2015) learned the sub-sequence representations from the raw protein sequences, and Doc2Vec (Yang et al., 2018) is proposed to use the full length of the protein sequence specifically for protein characteristic prediction. These methods fail to learn universal representations for protein sequences and have not been comprehensively collected for protein informatics (e.g., structural information and other relevant features). Additionally, a bidirectional LSTM (BiLSTM) model is proposed to learn embedding of protein sequences from structural informatics, by combining global structural similarity with the paired residue contacts of proteins (Bepler and Berger, 2019). Additionally, UniRep (Alley et al., 2019) used a Multiplicative-LSTM model to learn semantically rich representations from a massive protein sequence dataset. These approaches are not able to capture a longer range of information and is inefficient. More recently, pre-training language models such as BERT (Devlin et al., 2018) have shown great success in natural language processing (NLP), these models can learn contextualized word embedding with a large amount of available unlabeled text data and can achieve state-of-the-art performance in many language understanding tasks. Intuitively, there is potential in applying BERT to learn from protein sequences for the prediction of missense mutations.

In this paper, we propose a novel framework named BertVS (**Bert** for **v**ariant **s**equences classification) that predicts the pathogenicity of gene mutations. In particular, our proposed framework generally consists of three components. In the first component, BERT is pre-trained in the protein domain sequence from Pfam (Punta et al., 2000) with some preprocessing. In the second component, the protein mutation sequences are jointly represented by the pre-training BERT model and the amino acid hydrophilicity encoder. Finally, the classifier is trained for binary classification of protein mutation sequences in the last component. To the best of our knowledge, this is the first study to predict the missense mutation with a pre-training contextual language model. Compared with existing sequence-based models, our method achieves the best performance on two datasets, without prior knowledge of genetic variants. Moreover, we further perform experimental verification with clinical data on the ClinVar dataset. Additionally, it also shows that BertVS can be extended to almost all VUSs in the coding region. More importantly, we can observe from a series of systematic experiments that our predicted results are highly consistent with the analysis of experimental reports and other functional results.

## 2. Datasets

### 2.1. Gene Mutation Datasets

As we know, the BRCA1 gene has great influence on HDR, which is critical for tumor suppression. Saturation genome editing (SGE) (Findlay et al., 2018) is proposed to measure the functional effects of 3,893 SNVs in BRCA1 and whether these SNVs have been observed in humans. These verified SNVs are divided into three categories, including functional, non-functional, and the intermediate between them. In this paper, we focus on the influence of SNVs in the coding region of BRCA1. Specifically, we preprocess to exclude the specific SNVs that belong to the intermediate category. After that, we obtain 1,823 SNVs in BRCT which is a structural region with definite functional significance in BRCA1 (see Supplementary Table 1). We regard the corresponding protein sequence of 1,823 SNVs as sample data. In this paper, we only consider two types of SNVs (i.e., functional and non-functional). In reality, they are also referred to as benign and pathogenic mutations, respectively. We then use 1,823 mutation samples to train our proposed model. Among them, we take 392 pathogenic mutations as positive samples while the rest are benign mutations as negative samples. Further, we adopt the augmentation method to add terminators as noise to the sequences, due to the imbalance distribution of samples in classification task.

### 2.2. Protein Sequence Database

The non-synonymous single nucleotide substitutions (nsSNP) will result in the substitutions of amino acids, which can change the function and structure of the corresponding protein. Therefore, effectively exploring the relationship between protein function and structure has received much attention in recent years. Proteins can be expressed in a 3D structure with complex information, while these protein data are hardly available in most cases.

In this paper, we capture the contextual information of the protein sequence using unsupervised learning. As a large protein family database, Pfam (Punta et al., 2000) is selected as the corpus for pre-training the BERT model. In particular, we downloaded the FASTA files from Pfam and constructed a corpus with a total of 16,382 sequences by a keyword (i.e., BRCA1) filtering operation. We then preprocess each amino acid as a word and each sequence as a sentence. Next, we build a 20-word dictionary where each alphabet represents the corresponding type of amino acid. Table 1 shows the statistics of BRCA1-related domain data from the Pfam dataset. The identification number and protein family name are denoted by Accession and ID, respectively. For example, PF00533 is the identification number of *Accession*, and BRCT represents the name of the protein family.

**Table 1:** The statistics of BRCA1-related domain data from Pfam.

**Accession**	**ID**	**Description**
PF00533	BRCT	BRCA1 C Terminus (BRCT) domain
PF14835	zf-RING_6	zf-RING of BARD1-type protein
PF06209	COBRA1	Cofactor of BRCA1 (COBRA1)
PF12820	BRCT_assoc	Serine-rich domain associated with BRCT

## 3. Methods

In this section, we first provide an overview of the proposed BertVS (section 3.1). We then introduce the BERT pre-training model for protein sequence representation and encoding for amino acid hydrophilicity, respectively (sections 3.2–3.3). Finally, we discuss the mutation sequence prediction with our proposed model (section 3.4).

### 3.1. Overall of BertVS

**Figure 1:**
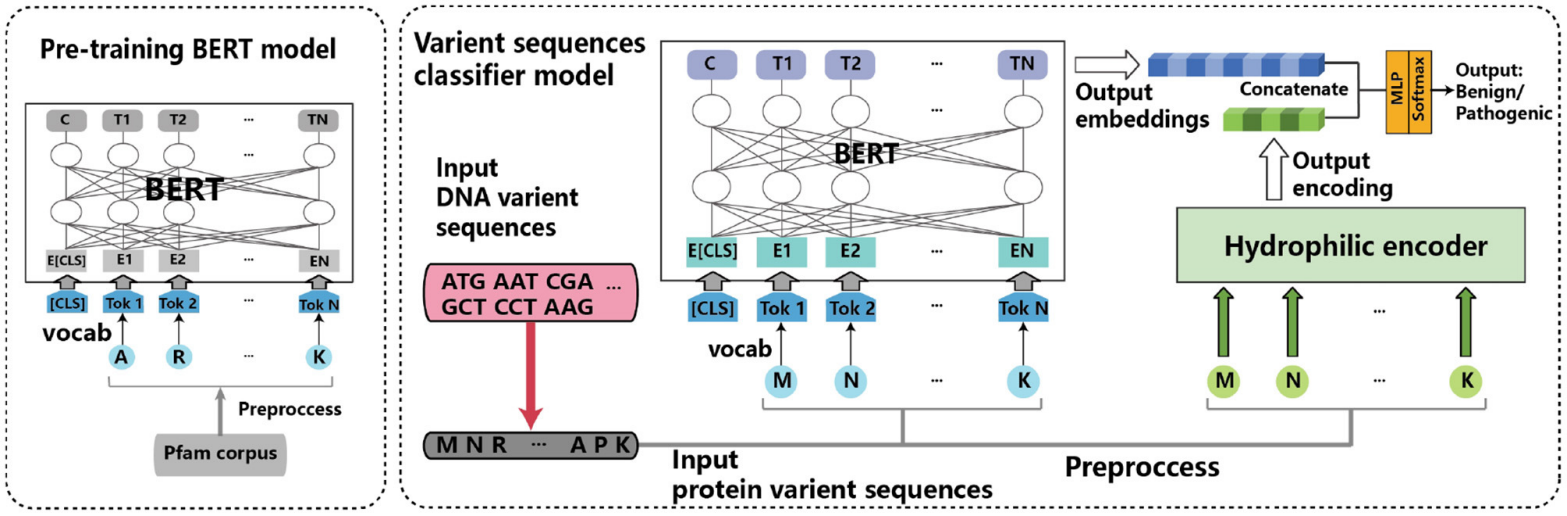
The overview of our proposed BertVS.

Figure 1 shows the overview of BertVS. It takes the symbolic sequences of the protein translated by DNA variant as the input, and outputs the prediction type of mutation sequences. Keep in mind that the central idea of BertVS is to consider both representation of protein sequence and encoding of thee hydrophilic property, by using a pre-training BERT model and embedding technique to encode the amino acids to a distributed representation. We therefore develop BertVS as a three-step framework for mutation sequence prediction:

1. Encoding symbolic tokens in protein domain sequences for pre-training;
2. Encoding the protein mutation sequences as well as the amino acids hydrophilicity;
3. Predicting the type of mutation sequences based on the encodings of the protein sequences and the hydrophilicity of amino acids.

Motivated by BERT (Devlin et al., 2018), in **the first step** in particular, we encode the symbols in the sequence of the protein to an embedding representation, using the BERT pre-training model. The sequence is converted into a vector through this step. In **the second step**, we extract features from the protein sequence, by encoding the protein mutation sequence and the hydrophilicity of amino acids. For protein sequence embedding, we consider the context of amino acids by protein mutation samples to fine-tune the BERT model. For the hydrophilic property of amino acids, we represent the unique biochemical properties using an encoder. As a result, we obtain two latent representations for the protein sequence containing the contextual information and the hydrophilic property, respectively. To predict the type of mutation sequence, in **the third step** BertVS inputs the concatenation of the two latent representations to a multi-layer perception classifier, and outputs a real value of mutation type. Next, we present the details of our proposed method.

### 3.2. BERT Pre-training Model

As a well-known language model, BERT has shown state-of-the-art performance in most natural language processing tasks (Devlin et al., 2018). Recently, there has been an increased interest in applying the BERT model to improve results in bioinformatics related tasks. Intuitively, we use a pre-training BERT_*BASE*_ model to generate the embedding for protein sequence representation learning. As a multi-layer bidirectional Transformer encoder, the BERT_*BASE*_ model contains 12 Transformer blocks, 768 hidden units, and 12 self-attention heads. The attention function can be constructed as follows:

(1) $$\begin{array}{l} Attention\left(Q,K,V\right)=Softmax\left(\frac{QK^{T}}{\sqrt{d_{k}}}\right)V \end{array}$$

where *Q*, *K*, and *V* are defined as the matrix of queries, keys, and values, respectively. *d*_*k*_ is the dimension of queries and keys. Instead of performing a single attention function, Multi-head attention (Vaswani et al., 2017) linearly projects the queries, keys, and values to *d*_*k*_, *d*_*k*_ and *d*_*v*_ dimensions, respectively. It can be described as follows:

(2) $$\begin{array}{l} MultiHead\left(Q,K,V\right)=Concate\left(head_{1},...,head_{h}\right)W^{O} \end{array}$$

where $head_{i}=Attention\left(QW_{i}^{Q},KW_{i}^{K},VW_{i}^{V}\right)$, *h* is the number of linear projections, and $W_{i}^{Q}\in {ℜ}^{d_{model}\times d_{q}}$, $W_{i}^{K}\in {ℜ}^{d_{model}\times d_{k}}$, $W_{i}^{V}\in {ℜ}^{d_{model}\times d_{v}}$ and $W^{O}\in {ℜ}^{hd_{v}\times d_{model}}$ are the projection parameter matrices.

In general, we input the preprocessed protein sequences into the model, and then it outputs the fixed embedding vector after training. Specifically, we first construct a protein sequence corpus from the Pfam dataset, and we then separate each amino acid by space as a word and assign the corresponding index to create a vocabulary. Furthermore, each protein sequence is separated by blank lines as a paragraph. All processed protein sequences are collected into a file as an input to the BERT model. Before the pre-training, BERT will further automatically preprocess the input data. After several epochs of training, the trained BERT model can learn the protein sequence representation by mapping the contextual information into the embedding vector. The output of the BERT pre-training model is the protein sequence embedding. Regardless of the input length of the protein sequence, we obtain the embedding vector of a fixed dimension and set the dimension to 768–equal to the hidden size of BERT.

### 3.3. Amino Acid Hydrophilicity Encoding

As an important property of amino acids, the hydrophilicity has a significant effect on protein function (Tan et al., 2019; Yang et al., 2019; Fu et al., 2020; Liu et al., 2020). For example, in c.5104-c.5112 (NCBI, NM_007294.3), Y1703 and F1704 of BRCA1 variants were scored as non-functional missense SNVs due to their hydrophobicity and internal position (Shiozaki et al., 2004; Findlay et al., 2018). Therefore, we consider the hydrophobicity of amino acids and integrate it into our model, which has a critical influence on the factor of variant function. Specifically, the amino acid in protein sequences are encoded by the hydrophilic value (Arias and Kyte, 1979). Table 2 shows the scale of hydropathical value among amino acids. We can see from Table 2 that higher positive values are more hydrophobic (e.g., Ile = 4.500), while lower negative values are more hydrophilic (e.g., Arg = −4.500). We then map each sequence to a distributed embedding, based on the corresponding hydrophilic value of amino acid *a*_*i*_:

(3) $$\begin{array}{l} f:X\left(a_{1},...,a_{i}\right)\to Hy_{x} \end{array}$$

where *f* is a mapping function, *X*(·) is the amino acid representation of the sequence *a*_1_, ..., *a*_*i*_, and *Hy*_*x*_ is the hydrophilic encoding matrix.

**Table 2:** The scale of hydropathical value among amino acids.

**Amino acid**	**Hydropathicity value**	**Amino acid**	**Hydropathicity value**
Ala	1.800	Leu	3.800
Arg	−4.500	Lys	−3.900
Asn	−3.500	Met	1.900
Asp	−3.500	Phe	2.800
Cys	2.500	Pro	−1.600
Gln	−3.500	Ser	−0.800
Glu	−3.500	Thr	−0.700
Gly	−0.400	Trp	−0.900
His	−3.200	Tyr	−1.300
Ile	4.500	Val	4.200

A fixed-size matrix is required for input into the encoder model, while the length of the protein sequence may vary. One simple solution is to fix the length of the input sequence in the dataset and to apply zero-paddings at the end of the input sequences when it less than the fixed size. We set the maximum length of the hydrophilicity vector to be the same as the maximum sequence length of the BERT model. The experimental results show that different sequence lengths have no effect on our model, as illustrated in section 4.2.3.

### 3.4. MLP Classifier

In this study, we look at mutation sequence prediction as a binary classification task by predicting the output value of the classifier. With the representation learned from the previous sections, we can integrate all the information from the protein sequence representation and the encoding hydrophilic value to predict the type of mutation sequence. In brief, we concatenate all representations and feed them to a multi-layer perception (Gardner and Dorling, 1998) to output the binary value. In the first hidden layer of the MLP, we compute the non-linear embedding of the sequence features extracted by BertVS:

(4) $$\begin{array}{l} h_{input}=\sigma \left(W^{1}Concate\left(Bert_{x},Hy_{x}\right)+b^{1}\right) \end{array}$$

where $Bert_{x}\in {ℜ}^{d_{BERT}\times n}$ and $Hy_{x}\in {ℜ}^{d_{Hy}\times n}$ are the matrices for the sequence representation of the BERT pre-training model and the hydrophilic encoder, respectively. σ(·) stands for a ReLU activation function over a single-layer neural network that is parameterized by the weight *W*^1^ and is a bias term *b*^1^. Furthermore, *d*_*BERT*_ and *d*_*Hy*_ denote the dimension of the hidden size of BERT and the maximum length of the hydrophilicity vector, respectively. Given a set of mutation sequences and the ground-truth values in the training dataset, we can use the binary cross-entropy as the loss function as follows.

(5) $$\begin{array}{l} l_{prediction}=-\left(ylogŷ\right)+\left(1-y\right)log\left(1-ŷ\right) \end{array}$$

where ŷ is the predicted value, *y* is the ground-truth value, which represents the actual label of type of mutation sequence (i.e., benign and pathogenic mutation).

## 4. Results

In this section, we first describe the experimental settings (section 4.1). Then, we compare our proposed method with state-of-the art models (section 4.2.1). We also conducted more experiments to analyze our model, including the classification performance and ablation study (section 4.2.2) to investigate the effectiveness of the main strategies adopted in this paper.

### 4.1. Experimental Settings

To evaluate the performance of BertVS in predicting the missense mutation, we randomly divided samples into three subsets with a ratio of 5/4/1, including fine-tuning, training, and testing sets. Next, we split the equal proportion of samples from the positive and negative samples as the test set, respectively. The noise sequences are added into the training set. Positive samples are labeled as 1 while negative samples are labeled as 0. The training epoch is set to 300, the learning rate is 0.001, and the weight-decay is empirically set to be 0.005 to prevent over-fitting. We use common metrics to evaluate the performance of our proposed method, including Accuracy, Recall, Precision, F1-score, AUROC, and AUPR. Note that AUROC denotes the area under Receiver operating characteristic (ROC) curve, and AUPR represents the area under Precision-recall (PR) curve.

To prove the performance of BertVS^1^, we compared it with state-of-the-art methods.

- **BiLSTM**: Bepler et al. (Bepler and Berger, 2019) learned protein sequence embeddings using information from structure. They trained a bidirectional long short-term memory (BiLSTM) as the pre-training model. The language model is pre-trained on the raw protein sequences in the Pfam to predict the amino acid at each position of each protein, given the previous amino acids and the following amino acids.
- **UniRep**: A Multiplicative-LSTM model learns statistical representations of proteins from 24 million UniRef50 sequences (Suzek et al., 2007). Without structural or evolutionary data, the unified representation (UniRep) summarizes arbitrary protein sequences into fixed-length vectors, which can approximate the fundamental protein features (Alley et al., 2019).
- **ProtVec**: A protein sequence representation and feature extraction method, which separates the sequences into sub-sequences to train distributed representations. It trains the embedding of sequences from Swiss-Prot through a Skip-gram neural network (Asgari and Mofrad, 2015).

For the comparison with BiLSTM, we modified the dimensions of the input embedding layer to the size of our model, and BiLSTM generates 21-dimension embeddings for each protein sequence. We used UniRep on our dataset and generated an embedding size of 1,900 for each protein sequence. Sequences with terminators are represented by a zero vector. For ProtVec, we generated the embedding of 300-dimensions for each sequence, and the sequences with terminators were treated in the same way as UniRep. The size of the hidden layer of the classifier is determined by the output dimension of the above method. The other parameter settings were kept the same as in the original work.

For the pre-training model, we need to train our BERT model from scratch. Our implementation utilizes the official code released by Microsoft for BERT model initialization. The maximum length of input sequence is set to 256, which results in 4,096 words per iteration on the basis of production of the maximum sequence length and the mini-batch size. Vocabulary includes a terminator symbol in addition to 20 amino acid abbreviations. It takes nearly 20 h for training with 20,000 rounds on a GPU of NVIDIA GP102, and we generated the embedding from the pre-training model of the amino acid sequence in the structural region of BRCA1. The predefined ratio of mutation data was used to fine-tune the model.

### 4.2. Experimental Results

#### 4.2.1. Comparison With Other Methods

**Figure 2:**
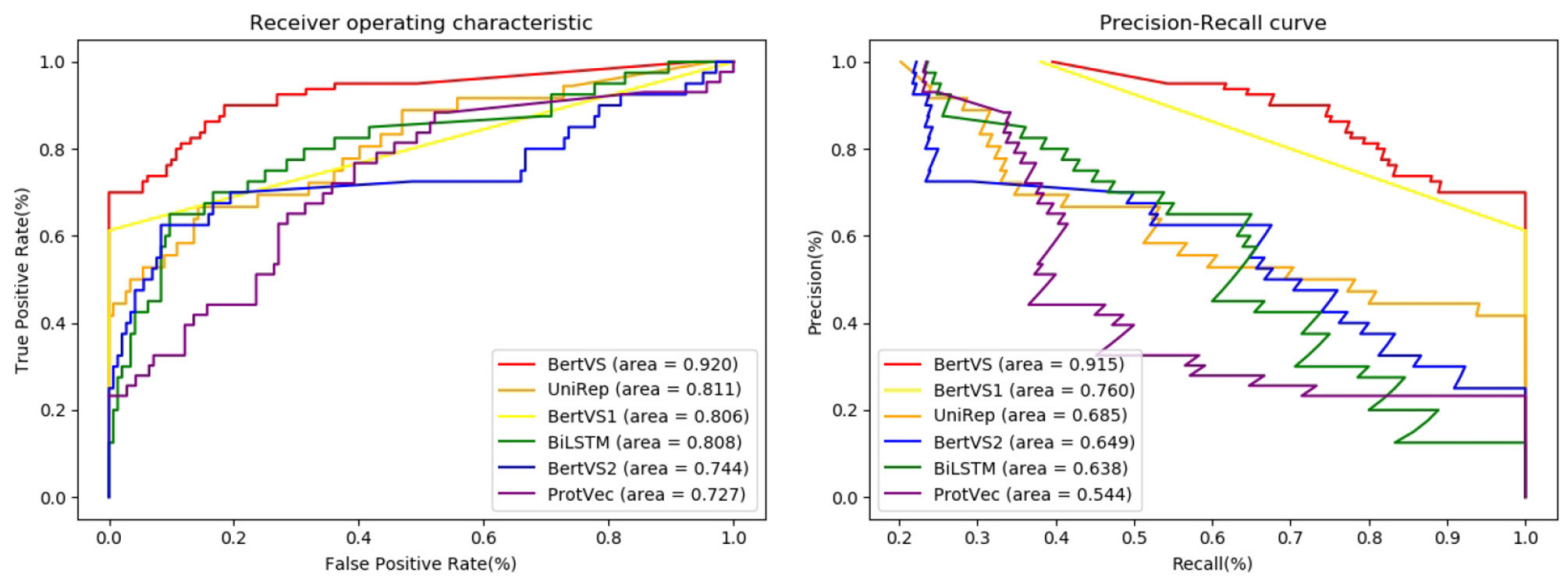
Predictive performance of different methods on the BRCT dataset. **Left:** Receiver operating characteristic (ROC) curves of prediction results obtained by applying BertVS and other comparison methods. **Right:** Precision-recall (PR) curves for BertVS and other methods. Note that BertVS1 means our model in feature-based mode, while BertVS2 means our model without pre-training model.

We conducted comparative experiments on 1,823 protein mutation samples. By setting different thresholds, we obtained the true positive rates (TPR) and false positive rates (FPR). As shown in Figure 2, the receiver-operating characteristics (ROC) curves were plotted by plotting TPR vs. FPR at different thresholds, where the area under receiver operating characteristics(AUROC) curve is used to evaluate the prediction performance of the proposed methods. From this observation, we found that BertVS achieved a value of 0.920 on AUROC, which significantly outperformed the value of UniRep (0.811). Figure 2 presents the Precision-Recall curves of different methods. Compared with the state-of-the-art model, BertVS also obtained superior performance (e.g., 23% improvement of AUPR). Furthermore, Table 3 shows the metrics of Accuracy, Recall, Precision, F1-score, AUROC, and AUPR of all comparison models, respectively. *Recall* measured the proportion of true positives recovered for the total number of them in the test set, and *Precision* metric is the fraction of true positives in the predictions. We found that BertVS achieved better results on precision. This shows that the positives retrieved by BertVS are all true positives. For a dataset with unbalanced positive and negative samples, the metric of *F*1-score can better reflect the prediction performance of the model. The *Recall*, *Precision*, and *F*1-score are formulated as shown below:

(6) $$\begin{array}{l} Recall=\frac{TP}{TP+FN} \end{array}$$

(7) $$\begin{array}{l} Precision=\frac{TP}{TP+FP} \end{array}$$

(8) $$\begin{array}{l} F1=\frac{2*Recall*Precision}{Recall+Precision} \end{array}$$

**Table 3:** The comparison results of all models on BRCA1 and PTEN datasets.

**Model/ Dataset**	**Accuracy**	**AUROC**	**AUPR**	**Recall**	**Precision**	**F1-score**
BiLSTM	0.826	0.808	0.638	0.500	0.625	0.556
UniRep	0.880	0.811	0.685	0.389	1.000	0.560
ProtVec	0.820	0.727	0.544	0.233	1.000	0.377
BertVS1	0.852	0.806	0.760	0.613	1.000	0.760
BertVS2	0.853	0.744	0.649	0.475	0.760	0.585
BertVS	0.857	0.920	0.915	0.625	1.000	0.769
Dataset (ClinVar_BRCA1)	0.890	0.898	0.717	0.861	0.778	0.815
Dataset (ClinVar_PTEN)	0.853	0.909	0.958	0.875	0.884	0.879

**Figure 3:**
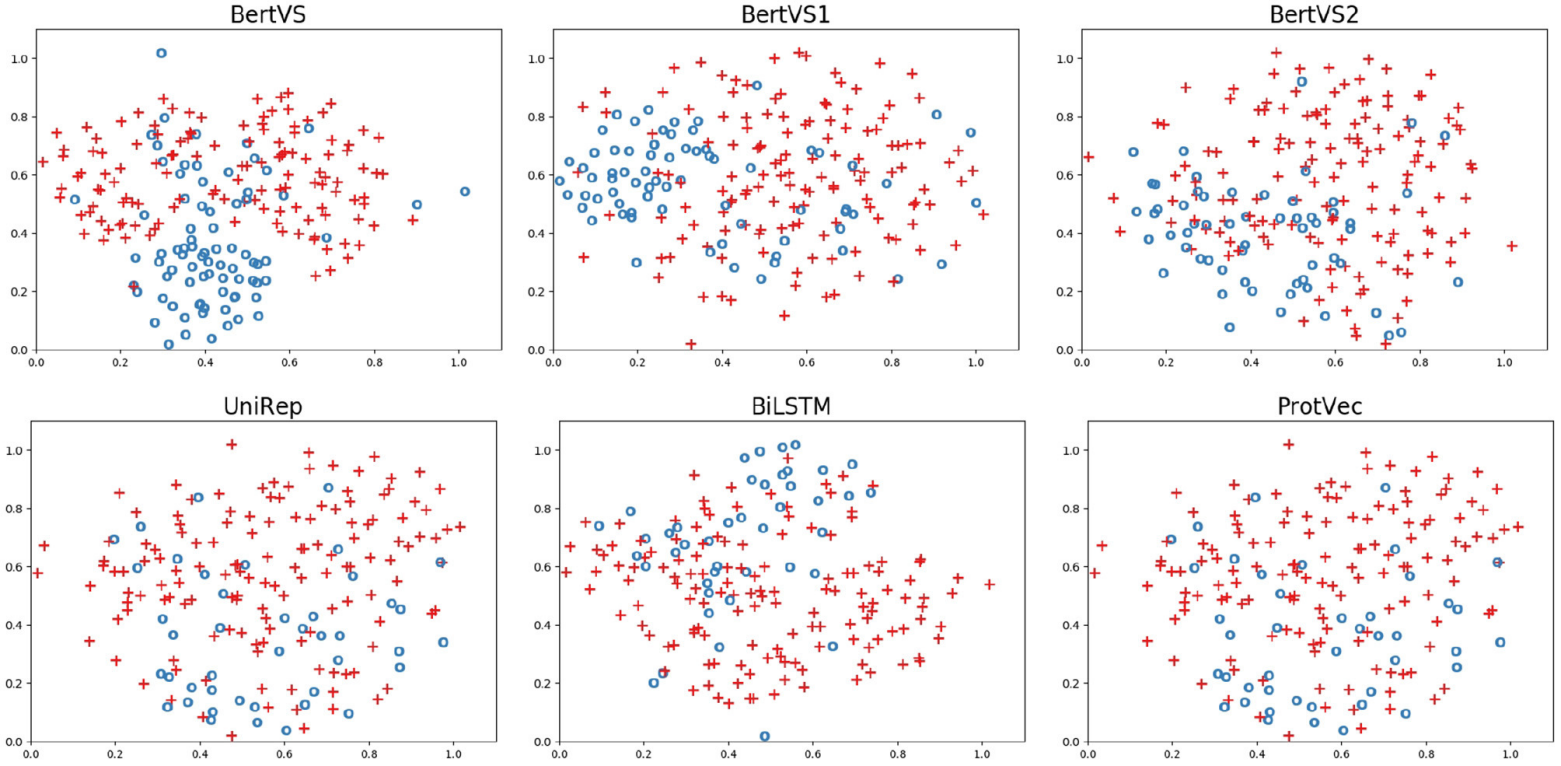
The two-dimensional representation of the protein mutation sequences learned by the t-SNE method. The red node represents a benign mutation, and the blue represents a pathogenic mutation.

Here, BertVS achieved at least 20.9% on the F1-score, a higher performance than UniRep (the second-best method). Our model and comparison methods are able to encode amino acids as the vector representation of the protein mutation sequence. The sequence representations obtained by different methods are visualized to assess their quality. We used t-SE (t-distributed Stochastic Neighbor Embedding) for visualization, which is a non-linear dimensionality reduction method that embeds similar points in the high-dimensional space into points close in two dimensions (Der Maaten and Hinton, 2008). The mutation sequence representations of the testing set were obtained by the trained models. The vector representation of each mutation sequence is mapped to a node in Figure 3, and the red node represents a benign mutation, and the blue represents a pathogenic mutation. Figure 3 shows that our model successfully performs the clustering of protein sequences in comparison to other methods. The reason could be that (i) compared to other methods, the corpus of BertVS is more targeted to the BRCA1 gene; and (ii) our model utilizes the biochemical properties of amino acids, which can better predict the mutations.

#### 4.2.2. Ablation Study

BertVS mainly contains two parts, that is, BERT for pre-training and amino acid hydrophilicity encoding. To examine the contribution of each component, we compared BertVS with several combinations. We implemented variants of our model, called BertVS1 and BertVS2. BertVS1 excludes the amino acid hydrophilicity encoding component and uses only the BERT pre-training model for classification. BertVS2 removes the pre-training component and replaces the BERT model with an embedding layer for sequence representation. The detailed configurations of the variant models are shown in Table 4. In the above experiments, the ratio of the training set to the test set becomes 9/1 and other experimental settings are the same as described in section 4.1. As shown in Table 3, we found that BertVS outperforms other variants in all metrics. Compared with BertVS1, which only considers the BERT pre-training model, BertVS is 11.4% and 15.5% higher on the metrics of AUROC and AUPR, respectively. This demonstrates that amino acid hydrophilicity encoding is beneficial in improving the mutation prediction performance. Moreover, BertVS achieves a AUROC score of 0.920 with about 17.6% improvement compared to BertVS2. The importance of the pre-training models is self-evident. In summary, we consider that the BERT pre-training model, combined with amino acid hydrophilicity encoding, can effectively predict mutation categories.

**Table 4:** The detailed description of the variants of our model.

**Model**	**Protein sequence representation**
BertVS1	BERT
BertVS2	Embedding layer & Amino acid hydrophilic encoding
BertVS	BERT & Amino acid hydrophilic encoding

#### 4.2.3. Impact of the Maximum Length of the Input Sequence

In this section, we discuss the impact of setting a hyper-parameter on the model performance. The experiment is also performed on 1,823 protein mutation samples. Except for the specific hyper-parameter discussed below, other parameters and experimental settings are kept the same as in section 4.1.

**Figure 4:**
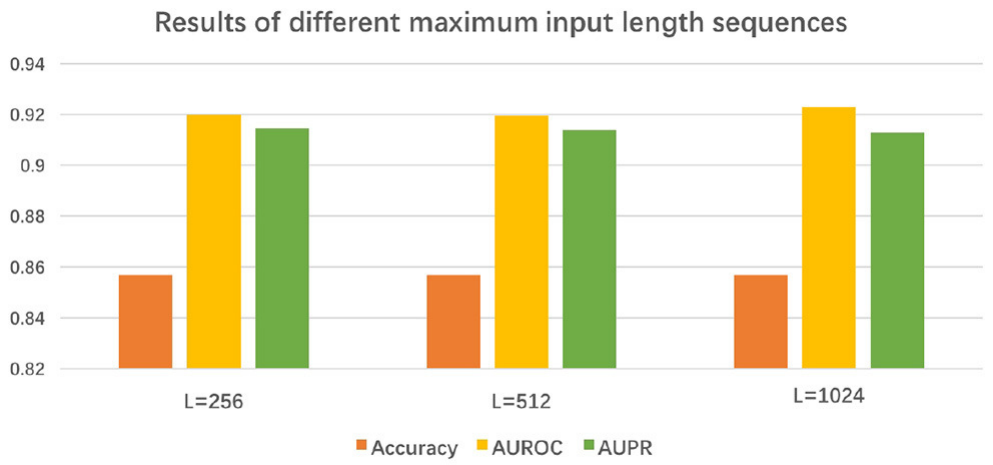
Results of BertVS with varying size of hyperparameter.

In order to process the input sequences of different lengths, we define the maximum length of the input sequence as L. We investigated the influence of L by varying it from 256 to 1024 for experiments. In Figure 4, we observe that the experimental results are only a marginal improvement. To save computing resources, we usually chose 256 as the maximum length of the input sequence.

#### 4.2.4. Performance of BertVS on the Clinical Dataset

**Figure 5:**
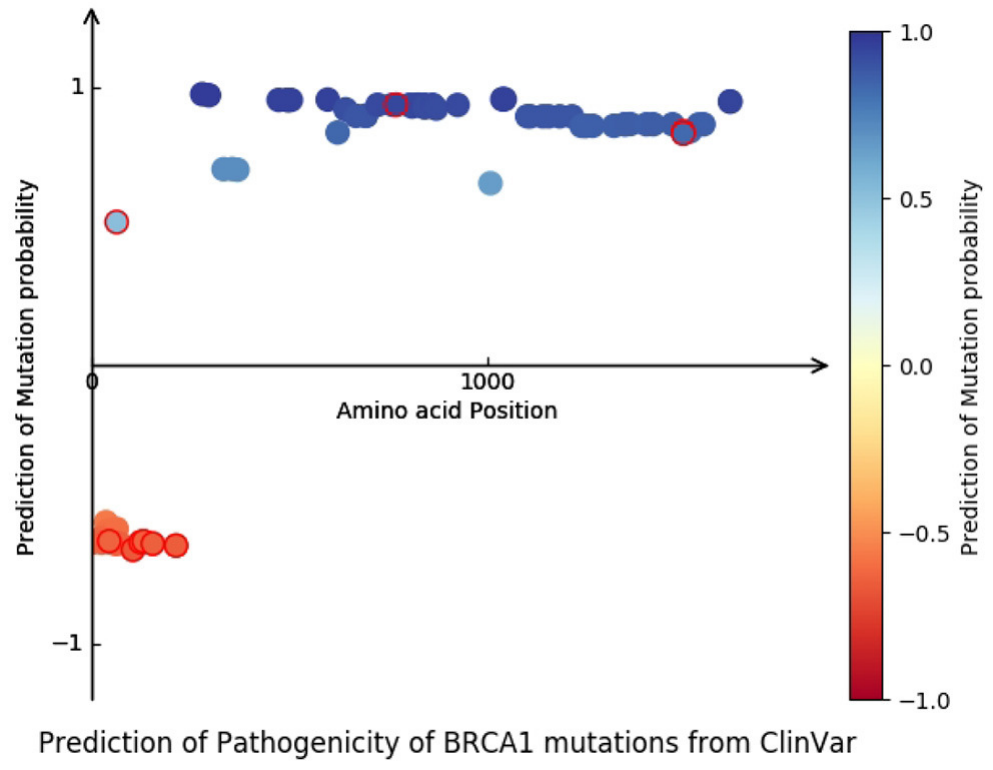
Predictive performance of BertVS on the BRCA1 dataset from ClinVar. The distribution of predicted probabilities and locations of BRCA1 mutations in ClinVar. The x-axis is the mutation position, and the y-axis is the predicted probability of the mutation.

Challenging and realistic scenarios are considered in our tests to evaluate the prediction performance of BertVS. Table 5 shows the variants that are predicted to be approximately pathogenic. We used 1,823 SNVs to train the BertVS and to predict other BRCA1 variants from the ClinVar database. In ClinVar database, 21 “Pathogenic” and 61 “Benign” of the BRCA1 missense mutations were selected, a total of 82 mutations as the test set. Conditions of these mutations include Breast and ovarian cancer (Hall et al., 1990), Hereditary cancer-predisposing syndrome (Frank, 2001), and FANCONI ANEMIA (Gluckman, 1989). To cross-validate clinical samples, we randomly divided the clinical data into two parts, one of which is added to the training set while the other is used as the test set. The prediction results on two-fold cross validation are shown in Table 3. As shown in Figure 5, the plot shows the predicted probability and location distribution of BRCA1 mutations from ClinVar. We observed that most of the pathogenic mutations distributes near the promoter of the gene. Furthermore, we used BertVS to predict missense VUSs, which are labeled “Likely benign” and “Likely pathogenic” from ClinVar in the BRCA1 gene.

**Table 5:** Prediction results of missense VUSs of BRCA1.

**Name**	**Protein change**	**Clinical significance**	**Pathogenicity**
c.5566C>T (p.Pro1856Ser)	P1856S, P1809S, P752S, P1877S	Likely benign(Last reviewed: May 3, 2018)	0.997
c.2230G>A (p.Ala744Thr)	A744T, A697T	Likely benign (Last reviewed: Jun 29, 2016)	0.938
c.2207A>G (p.Glu736Gly)	E736G, E689G	Likely benign (Last reviewed: Feb 15, 2016)	0.909
c.2374G>A (p.Gly792Arg)	G792R, G745R	Likely benign (Last reviewed: Jul 3, 2017)	0.853
c.5153G>C (p.Trp1718Ser)	W1718S, W1671S, W1739S, W614S	Likely pathogenic (Last reviewed: Aug 4, 2015)	0.847
c.2177T>C (p.Leu726Pro)	L726P, L679P	Likely benign (Last reviewed: Apr 18, 2017)	0.831
c.2083G>T (p.Asp695Tyr)	D695Y, D648Y	Likely benign (Last reviewed: Mar 5, 2019)	0.811
c.4726G>C (p.Glu1576Gln)	E1576Q, E1529Q, E1597Q, E472Q	Likely benign (Last reviewed: Nov 10, 2014)	0.783
c.4750G>T (p.Ala1584Ser)	A1584S, A480S, A1537S, A1605S	Likely benign (Last reviewed: Feb 22, 2019)	0.783
c.1912G>A (p.Glu638Lys)	E638K, E591K	Likely benign (Last reviewed: Apr 18, 2016)	0.782
c.1522C>G (p.Pro508Ala)	P508A, P461A	Likely benign (Last reviewed: Jul 18, 2016)	0.772
c.1390A>G (p.Thr464Ala)	T464A, T417A	Likely benign (Last reviewed: Apr 21, 2016)	0.763
c.1487G>T (p.Arg496Leu)	R496L, R449L	Likely benign (Last reviewed: Aug 25, 2016)	0.757
c.4328G>A (p.Arg1443Gln)	R1443Q, R1396Q, R340Q	Likely benign (Last reviewed: Dec 8, 2015)	0.738
c.4185G>C (p.Gln1395His)	Q1395H, Q292H, Q1348H	Likely pathogenic (Last reviewed: Dec 21, 2017)	0.736
c.4565A>G (p.Tyr1522Cys)	Y1522C, Y1543C, Y1475C, Y418C	Likely benign (Last reviewed: Jan 23, 2018)	0.696

**Figure 6:**
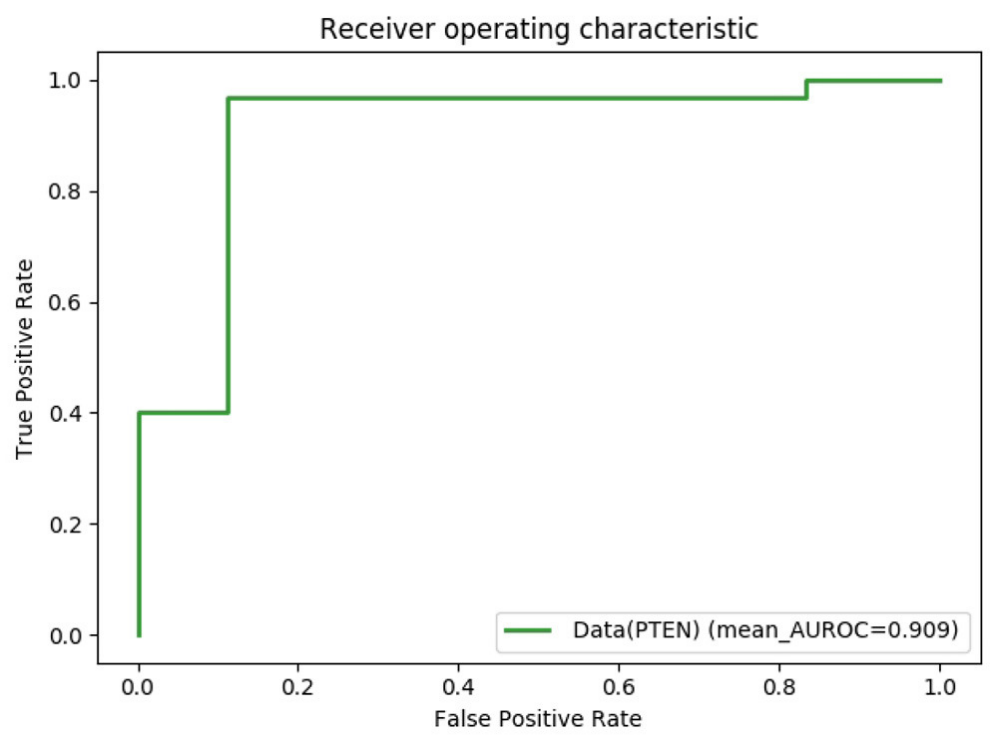
Predictive performance of BertVS on the PTEN dataset from ClinVar. Receiver operating characteristic (ROC) curves of prediction results obtained by applying BertVS.

In addition to the variants of the BRCA1 gene, we also experimentally test the variants of the PTEN gene. We expect our model to be equally effective in different genes under various biological mechanisms. We collected PTEN alignment sequences named PF10409 from the Pfam database, they are also described as C2 domain of PTEN tumor-suppressor protein. The dataset of PTEN mutation sequences seems scarcer and more unbalanced and is also collected and screened from ClinVar. Conditions of PTEN mutations include Cowden syndrome 1 (Pilarski, 2009), PTEN hamartoma tumor syndrome (Mester and Charis, 2016), Cutaneous melanoma (Bittner et al., 2000; Balch et al., 2001) etc. A total of 44 mutations, with only one negative (benign) sample, made data enhancement indispensable. Different from the BRCA1 variants dataset, PTEN needs to add negative samples as noise. The same length of normal PTEN protein sequences are truncated and added to the training set as negative samples. As shown in Figure 6, BertVS for variants of the PTEN gene achieved a great prediction performance. In summary, the above effects of our model further prove its strong predictive ability and future prospects in the clinical diagnosis and treatment of genetic mutations. In addition, the prediction results of PTEN missense mutations were labeled as “Likely benign” and “Likely pathogenic” from ClinVar.

## 5. Conclusion

In this paper, we propose a novel framework named BertVS to predict missense mutations. To our knowledge, we are the first to apply the BERT model to a representation learning of protein sequence to predict the pathogenicity of gene mutations. Specifically, we extracted the contextual information from the protein sequence as well as the hydrophilic property of an amino acid encoder. The experimental results illustrate the performance of our proposed method with comparison to baselines. Moreover, we also verify the superior performance of our method on clinical data. Our method has good robustness and shows good generalization performance on BRCA1 and PTEN gene datasets. For future research directions, computational intelligence such as neural networks (Song et al., 2017, 2020; Hong et al., 2020), evolutionary algorithms (Xu et al., 2017), and unsupervised learning (Zou et al., 2018; Zeng et al., 2019a), which have been applied in the prediction of drug targets (Quan et al., 2019; Zeng et al., 2019b; Lin et al., 2020b), disease related miRNAs (Zhang et al., 2017; Zeng et al., 2018), can be employed in this field.

## Data Availability Statement

The original contributions presented in the study are included in the article/Supplementary Material, further inquiries can be directed to the corresponding authors.

## Author Contributions

KL and YZ designed the study and wrote the manuscript. KL and XL translated manuscript. YZ analyzed data and drawn illustrations. ZQ provides theoretical guidance on protein mutation sequences. All authors have read and approved the final manuscript.

## Conflict of Interest

The authors declare that the research was conducted in the absence of any commercial or financial relationships that could be construed as a potential conflict of interest.

## Footnotes

^1^https://github.com/xzenglab/BertVS
