# Supplementary material for: Predicting the Disease Risk of Protein Mutation Sequences With Pre-training Model
Source: Front Genet. 2020 Dec 21;11:605620. doi: 10.3389/fgene.2020.605620 (PMC7780924; doi:10.3389/fgene.2020.605620)
Supplement: Supplementary file 1 [file Table_1.pdf]

Supplementary Material

Supplementary Table S1. Hyperparameters setting

Length	Accuracy	AUROC	AUPR	Recall	Precision	F1-score
256	0.8571	0.9199	0.9146	0.6250	1.0000	0.7692
512	0.8571	0.9193	0.9140	0.6250	1.0000	0.7692
1024	0.8571	0.9228	0.9127	0.6250	1.0000	0.7692
Unit	Accuracy	AUROC	AUPR	Recall	Precision	F1-score
64	0.8571	0.8938	0.9008	0.6250	1.0000	0.7692
128	0.8571	0.9199	0.9146	0.6250	1.0000	0.7692
256	0.8571	0.9186	0.9120	0.6250	1.0000	0.7692
512	0.8571	0.9191	0.9138	0.6250	1.0000	0.7692
1024	0.8571	0.9148	0.9083	0.6250	1.0000	0.7692

Note: AUROC: the area under ROC curve; AUPR: the area under PR curve.
